# The Association at Augusta

**Published:** 1896-05

**Authors:** 


### THE ASSOCIATION AT AUGUSTA.

In another place will be found a detailed report of the late meeting of our Association. While the meeting was perhaps not quite so well attended as others have been, it was highly successful, both from the character of work done, and for the recreation which it afforded the members. The address of welcome by Dr. Eugene Foster was in this learned gentleman's usual happy style. After reciting some of the objects and achievements of the Association, Dr. Foster reviewed some of our State medical history, mentioning the names of the men who have reflected distinction and honor upon our profession; such as Antony, Daniel, Dugas, Ford, Eve, Fort, Banks, Long, Campbell, Westmoreland, Battey, etc. He also congratulated the Association and the profession upon the final success of the efforts to obtain a good examining board. The response by Dr. E. R. Anthony, of Griffin, was eloquent and full of feeling.

The papers presented were fully equal to those usually read at these meetings. The paper by Dr. Samuel Lloyd, of New York, on "Appendicitis," was particularly good, calling forth considerable discussion. A good abstract of papers appears in this issue.

The thanks of the visiting members are heartily due the local profession for the elegant social features which contributed so much to their entertainment. The barbecue at the *Deutcher Scheutzen Platz* was a great success and highly enjoyed. So was the musicale, which was kindly furnished by some of the well-known musical talent of Augusta. The members are much indebted to the profession and the citizens of Augusta for a very pleasant stay in their city.

